# Diffuse cystic lung diseases—a primer for radiologists

**DOI:** 10.1093/bjro/tzag010

**Published:** 2026-05-12

**Authors:** Li Peng, Lan Song, Jinhua Wang, Kepei Xu, Helmut Prosch

**Affiliations:** Department of Radiology, State Key Laboratory of Complex Severe and Rare Diseases, Peking Union Medical College Hospital, Chinese Academy of Medical Sciences and Peking Union Medical College, Beijing 100730, China; Department of Radiology, State Key Laboratory of Complex Severe and Rare Diseases, Peking Union Medical College Hospital, Chinese Academy of Medical Sciences and Peking Union Medical College, Beijing 100730, China; Department of Radiology, State Key Laboratory of Complex Severe and Rare Diseases, Peking Union Medical College Hospital, Chinese Academy of Medical Sciences and Peking Union Medical College, Beijing 100730, China; Department of Radiology, State Key Laboratory of Complex Severe and Rare Diseases, Peking Union Medical College Hospital, Chinese Academy of Medical Sciences and Peking Union Medical College, Beijing 100730, China; Department of Biomedical Imaging and Image-guided Therapy, Medical University of Vienna, A-1090 Vienna, Austria

**Keywords:** cystic lung diseases, pulmonary cysts, imaging features, differential diagnosis, multidisciplinary collaboration

## Abstract

Diffuse cystic lung diseases (DCLDs) comprise a broad spectrum of disorders characterized by diffusely distributed pulmonary cysts, posing significant diagnostic challenges due to overlapping imaging features. High-resolution CT serves as the cornerstone for the diagnosis of DCLDs, yet its reliability is limited by technical constraints, including insufficient spatial resolution for subtle cysts and variable correlation with functional impairment. Furthermore, persistent discrepancies between imaging findings and underlying pathology highlight the inherent limitations of current modalities. This review synthesizes current diagnostic workflows, emphasizing the integration of imaging with clinical, genetic, and histopathological data within a multidisciplinary framework and appraising the impact of emerging technologies on diagnostic precision. Throughout this evolution, radiologists retain a central role in timely recognition, collaborative decision-making, and outcome optimization for patients with DCLDs.

## Introduction to the evolving landscape of diffuse cystic lung diseases

Diffuse cystic lung diseases (DCLDs) are a heterogeneous group of rare disorders characterized by pulmonary cyst formation, with emerging evidence suggesting a rising prevalence.^1^ Diagnosis is often difficult due to overlapping imaging features and nonspecific clinical presentations. Moreover, treatment, prognosis, and management strategies vary substantially across subtypes. Misinterpretation can delay appropriate intervention or prompt unnecessary invasive procedures, underscoring the need for a systematic, multidisciplinary approach that integrates multimodal clinical and imaging data to establish accurate diagnoses and guide precision-based management. High-resolution CT (HRCT) remains the cornerstone noninvasive modality for detecting and characterizing DCLDs, providing high spatial resolution, isotropic 3D imaging, and rapid acquisition. When combined with the broader clinical context, HRCT enables early diagnosis, longitudinal monitoring, and comprehensive disease assessment. This review synthesizes clinicoradiologic correlations and evidence-based HRCT interpretation frameworks to propose systematic diagnostic approaches, address the limitations of conventional imaging, and highlight emerging techniques, including advanced structural, functional, and molecular imaging, as well as artificial intelligence (AI), that promise to enhance diagnostic precision and facilitate individualized, precision-guided management.

## Etiological classification and imaging features of DCLDs

The etiology of DCLDs is multifactorial, involving genetic and environmental factors that generate both homogeneous and heterogeneous radiologic manifestations from the prenatal period through adulthood. A comprehensive etiological taxonomy is provided in Table 1.

**Table 1:** Etiological classification of cystic lung diseases.

**Neoplastic**	Lymphangioleiomyomatosis (sporadic and TSC-associated)
	Pulmonary Langerhans cell histiocytosis, and non-Langerhans cell histiocytoses (eg, Erdheim Chester disease)
	Primary and metastatic neoplasms (eg, sarcomas, adenocarcinomas, pleuropulmonary blastoma, and benign metastasizing leiomyoma)
**Genetic/developmental/congenital**	Folliculin deficiency syndrome (formerly called Birt-Hogg-Dubé syndrome)
	Proteus syndrome, neurofibromatosis, Ehlers-Danlos syndrome, Marfan syndrome
	Congenital pulmonary airway malformation, pulmonary sequestration, congenital lobar emphysema, bronchogenic cysts; bronchopulmonary dysplasia.
	Hyper IgE syndrome (Job syndrome)
**Associated with lymphoproliferative disorders**	Lymphoid interstitial pneumonia
	Follicular bronchiolitis
	Sjögren’s disease
	Amyloidosis
	Light chain deposition disease
	IgG4-related disease
	Lymphoma (eg, MALToma)
	Castleman disease
**Infectious**	*Pneumocystis jirovecii* pneumonia
	Staphylococcal pneumonia
	Respiratory papillomatosis
	Fungal diseases (eg, coccidioidomycosis)
	Parasitic diseases (eg, paragonimiasis)
**Associated with interstitial lung diseases**	Hypersensitivity pneumonitis
	Alveolar macrophage pneumonia (formerly called desquamative interstitial pneumonia)
**Smoking related**	Pulmonary Langerhans cell histiocytosis
	Respiratory bronchiolitis
	Alveolar macrophage pneumonia (formerly called desquamative interstitial pneumonia)
	Respiratory bronchiolitis
**Occupational/environmental**	Aluminum dust pneumoconiosis
	Fire-eater’s lung
	Hypersensitivity pneumonitis
	Hard metal lung disease (tungsten carbide sensation)
	Chronic beryllium disease (beryllium sensitization)
	Hut lung (biomass fuels)
**Mimics of cystic lung disease**	Bleb, bulla
	Bronchiectasis
	Cavity
	Cystic lung cancer
	Emphysema (eg, alpha1-antitrypsin deficiency)
	Honeycomb (eg, sarcoidosis, idiopathic pulmonary fibrosis)
	Post-traumatic pseudocysts/pneumatocele

Abbreviations: MALToma = mucosa-associated lymphoid tissue lymphoma; TSC = tuberous sclerosis complex.

### Key imaging features of the principal disorders

Cyst patterns, associated pulmonary findings, and relevant extrapulmonary clues of major diseases are summarized here to facilitate diagnosis.

#### Congenital pulmonary airway malformation

**Figure 1:**
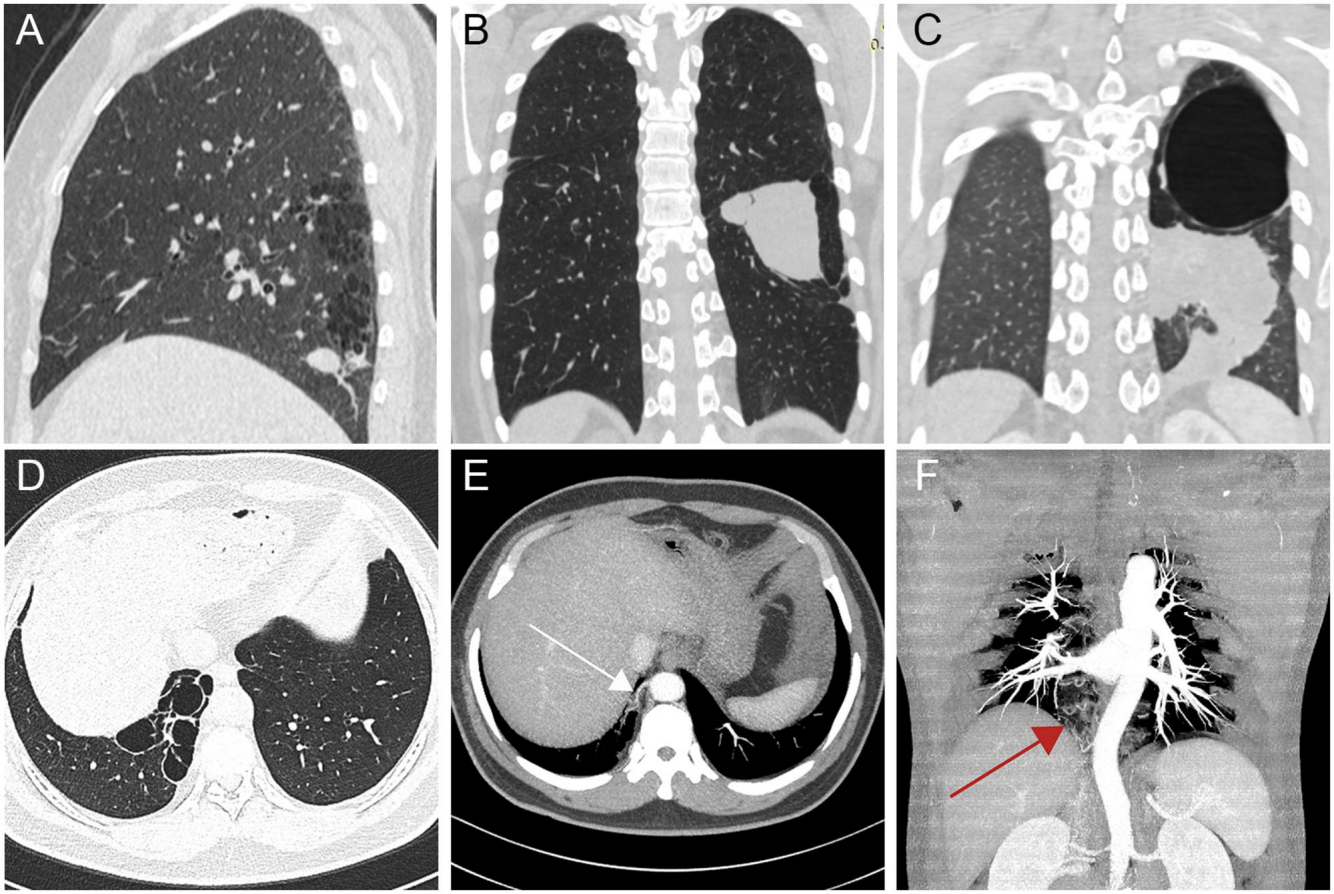
Congenital cystic lung diseases. CPAM: 63-year-old woman, multiple cysts with irregular soft-tissue mass in the right lower lobe (A); 25-year-old woman, oval soft-tissue mass in the left lower lobe (B); and 24-year-old woman, large irregular soft-tissue mass with giant pulmonary cyst in the left lung (C). PS in a 43-year-old man (D-F). Axial chest CT image (D) demonstrates a well-demarcated, air-filled, paravertebral multicystic lesion in the posterior segment of the right lower lobe. 3D reconstruction shows systemic arterial supply from a branch of the thoracic aorta (E, white arrow) and systemic venous drainage (F, red arrow). Abbreviations: CPAM = congenital pulmonary airway malformation; PS = pulmonary sequestration.

Congenital pulmonary airway malformation (CPAM), the most common congenital lung malformations (CLMs), affects roughly 1 in 7200-35 000 live births.^2^ Five histologic subtypes (0-4) exist, type 1 predominates, followed by type 2 and type 3.^3^ Radiologically, CPAM usually involves a single lobe-most often the lower lobe in adults-though multilobar involvement can occasionally occur (Figure 1A-C). Subtype-specific CT patterns: Type 0, diffusely hypoplastic lungs with innumerable tiny/microscopic cysts, showing a sponge-like or ground-glass radiologic appearance; Type 1, one or a few large (>3 cm), well-defined air-filled cysts, occasionally with air-fluid levels; Type 2, multiple small (<2 cm) cysts that may coalesce or appear as focal or ill-defined consolidations when below CT resolution; Type 3, bulky solid or microcystic masses causing mediastinal shift and ipsilateral lung hypoplasia; Type 4, large peripheral or complex cysts, sometimes bilateral/multifocal; can mimic cystic pleuropulmonary blastoma (PPB) and may present with pneumothorax.^4^^,^^5^  *DICER1* mutation raises concern for PPB. Secondary infection thickens cyst walls, produces air-fluid levels, and provokes perilesional inflammation. Pulmonary interstitial emphysema (PIE) and pneumatoceles rarely mimic CPAM.^6^ CT angiography excludes systemic arterial supply typical of pulmonary sequestration (PS) (Figure 1D-F), yet hybrid CPAM-PS lesions may coexist.

#### Lymphangioleiomyomatosis

**Figure 2:**
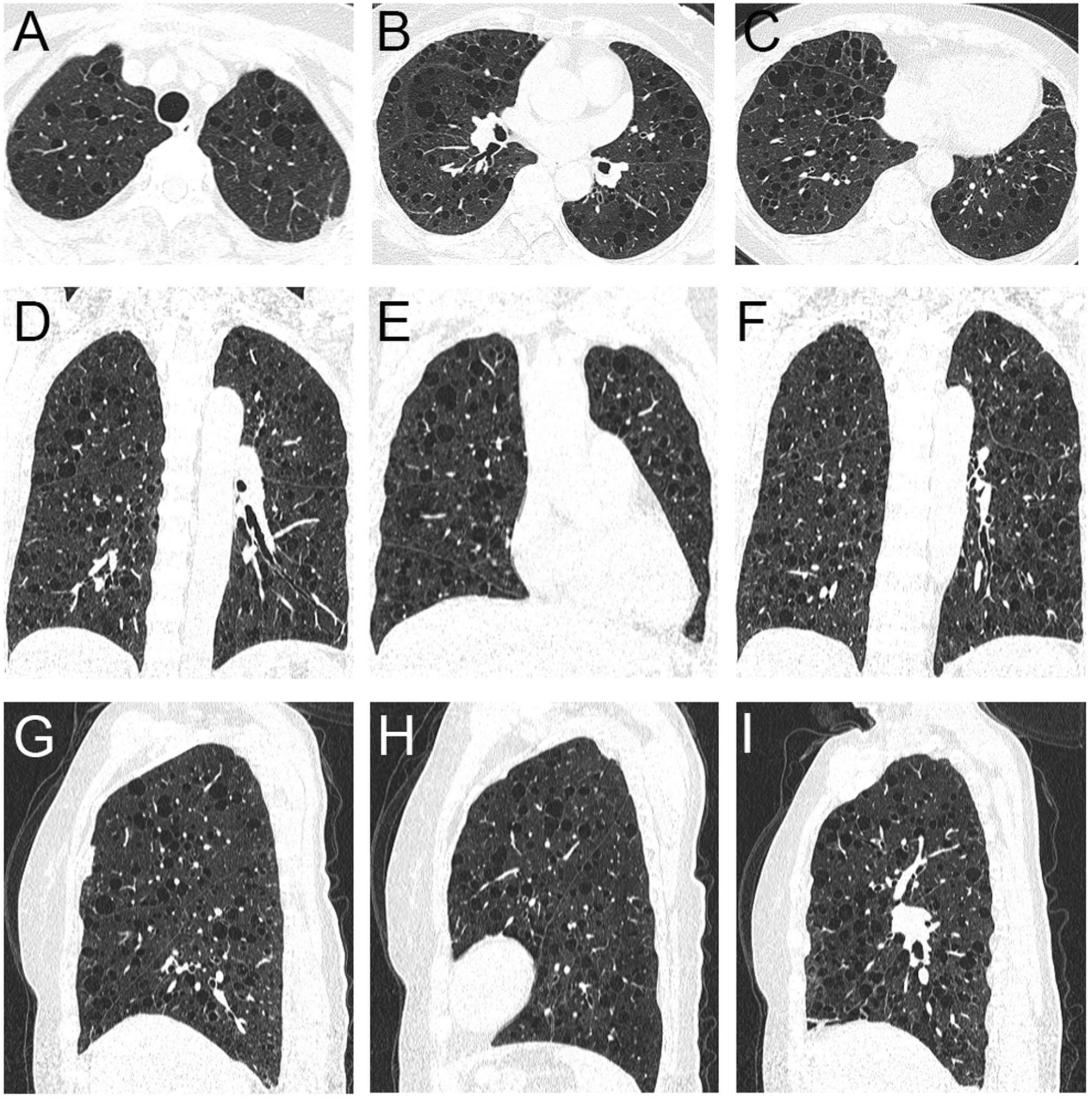
LAM in a 67-year-old woman. Axial (A-C), coronal (D-F), and sagittal (G-H) CT images demonstrate numerous smooth, thin-walled, round, or oval cysts evenly distributed across both lungs, including the costophrenic angles. The cysts lack internal septa or traversing vessels, and the intervening lung parenchyma appears normal. Abbreviation: LAM = lymphangioleiomyomatosis.

Lymphangioleiomyomatosis (LAM) is a rare, slowly progressive, multisystem neoplasm of low malignant potential in the perivascular epithelioid cell tumor (PEComa) family. It is characterized by proliferation of abnormal smooth muscle-like cells (LAM cells), leading to progressive cystic destruction of the lung, lymphatic abnormalities such as lymphangioleiomyomas and chylous effusions, or abdominal tumors including renal angiomyolipomas (AMLs). It arises from TSC1/TSC2 mutations with mTOR pathway hyperactivation.^7^ LAM occurs sporadically (S-LAM) or in association with tuberous sclerosis complex (TSC-LAM),^8^ almost exclusively in women—mainly premenopausal and typically diagnosed in their thirties—although cases range from adolescence to octogenarians and prevalence increases with age.^9^ Male LAM is exceptional and usually linked to TSC-related mosaicism or occult mutations.^9^ Radiologically, LAM is characterized by diffuse, symmetric, thin-walled cysts, typically 2-20 mm (occasionally >30 mm), well-circumscribed, round or ovoid, without septations or traversing vessels, and with normal intervening lung (Figure 2). Cysts involve all lobes, including costophrenic angles. Atypical features include large or coalescent cysts (mimicking emphysema or hydropneumothorax), irregular shapes, ground-glass opacities (GGOs), noncalcified nodules (2-14 mm, often representing multifocal micronodular pneumocyte hyperplasia), interlobular septal thickening, reticulation, wall thickening, or rare wall calcification.^10^^,^^11^ Extrapulmonary manifestations include spontaneous pneumothorax (SP), chylous pleural effusions, and pleural masses or calcifications. Extrathoracic manifestations include AMLs (renal, hepatic, rarely peritoneal), lymphangioleiomyomas (mainly retroperitoneal, occasionally pelvic or mediastinal), and lymphangiomas (retroperitoneal). Additional findings include ascites, thoracic duct dilatation, lymphadenopathy, and cervicomediastinal hamartoma. A definitive diagnosis of LAM requires a multimodal approach. The identification of characteristic thin-walled cysts on HRCT is necessary but insufficient alone.^12^^,^^13^ A diagnosis of definite LAM is established when these typical HRCT findings are present plus at least one of the following confirmatory features^12^: (1) pathological confirmation (via lung, lymph node, or mass biopsy); (2) presence of tuberous sclerosis complex (TSC); (3) extrapulmonary manifestations such as renal AML(s), lymphangioleiomyoma, or chylous (thoracic/abdominal) effusion; or (4) an elevated serum vascular endothelial growth factor-D (VEGF-D) level ≥800 pg/mL. Serum VEGF-D serves as a key biological diagnostic criterion, particularly for sporadic LAM.^12^^,^^13^ For patients with characteristic HRCT but lacking other clinical or radiographic confirmatory features, VEGF-D testing at this threshold is recommended to establish the diagnosis before considering invasive lung biopsy.^12^^,^^13^

#### Pulmonary Langerhans cell histiocytosis

**Figure 3:**
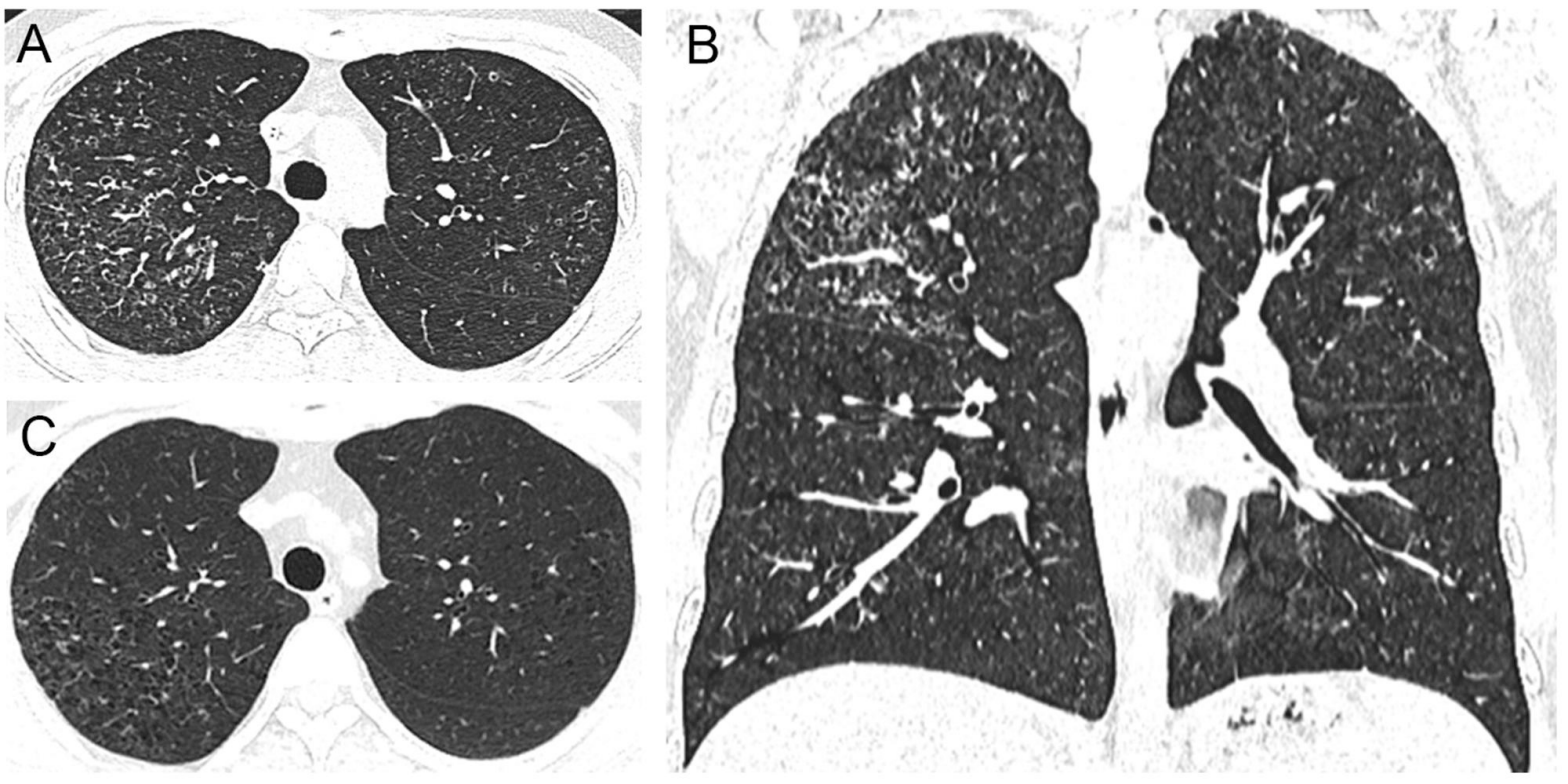
PLCH in a 32-year-old male smoker. (A, B) Baseline CT demonstrates multiple upper-lobe predominant cysts of varying wall thickness, scattered nodules, and bilateral GGOs. (C) Eight-month follow-up CT shows nodules and GGOs have nearly resolved, while most cysts persist. Abbreviations: PLCH = pulmonary langerhans cell histiocytosis; GGO = ground-glass opacity.

Pulmonary Langerhans cell histiocytosis (PLCH) is an inflammatory myeloid neoplasm driven by MAPK-pathway mutation. PLCH mainly affects young adults (20-40 years), with women presenting slightly later, and is strongly associated with smoking.^14^^,^^15^ Radiologically, early disease manifests as small, irregular, centrilobular/peribronchiolar nodules in a bilaterally symmetric upper- and mid-lung distribution, while characteristically sparing the lung bases and costophrenic angles. GGOs may coexist at this stage.^16^ Over time, the nodules may cavitate, forming thick-walled cysts (Cheerio sign). A subset of these cysts develops thin, linear septa that radiate from a central scar, creating the characteristic octopus sign.^17^ Later, these cysts thin and coalesce into bizarre-shaped cysts (typically <10 mm) in the same distribution; costophrenic angle sparing is typical in adults but is frequently absent in children. It is critical to note that pediatric PLCH is predominantly associated with multisystem disease, in contrast to the strong smoking-related etiology observed in adults.^18^ Cyst walls vary from imperceptible (mimicking emphysema) to thick, irregular, or nodular; superimposed infection/hemorrhage can simulate cavitary metastases or septic emboli.^19^ Intervening lung parenchyma is usually normal, whereas GGOs often reflects smoking-related co-morbidities. The dynamic coexistence of nodules and cystic lesions during disease progression provides a high-specificity imaging hallmark for diagnosing PLCH in young smokers. Acute-phase GGOs and nodules may regress, leaving residual cysts (Figure 3). Pneumothorax also occurs relatively commonly in PLCH patients. Importantly, PLCH may be associated with multisystem disease.

#### Folliculin deficiency syndrome

**Figure 4:**
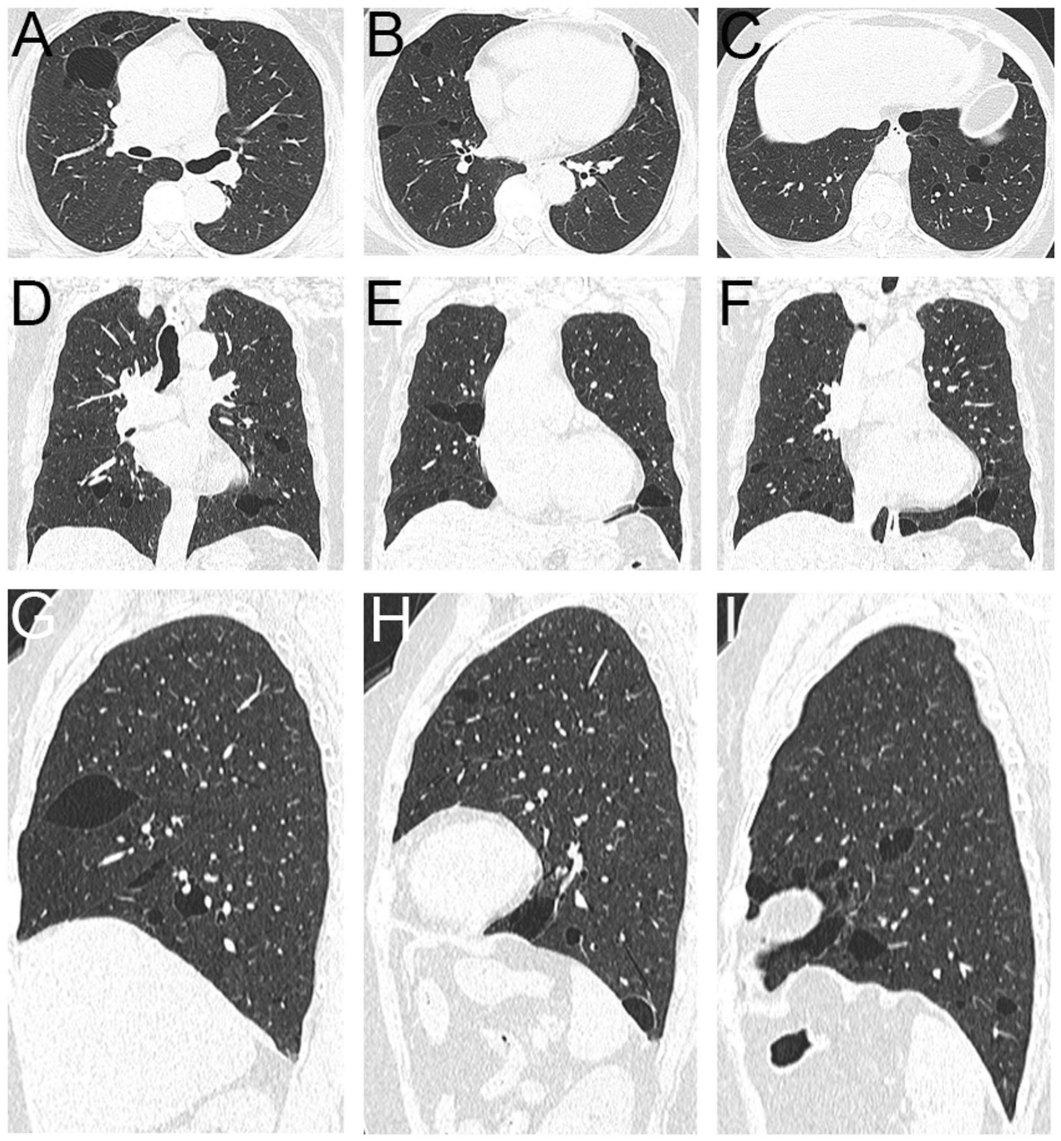
FDS in a 58-year-old woman. CT images (A-C, axial; D-F, coronal; G-H, sagittal) show multiple, well-defined, thin-walled pulmonary cysts of varying size and morphology—round, oval, and irregular—clustered in the medial, mid-lung, and lower lobes, with a characteristic subpleural distribution along the mediastinal pleura, interlobar fissures, and costophrenic angles. Abbreviation: FDS = folliculin deficiency syndrome.

Folliculin deficiency syndrome (FDS, formerly called Birt-Hogg-Dubé syndrome) is a rare autosomal-dominant disorder caused by germline FLCN variants, marked by pulmonary cysts (predisposing to SP), benign cutaneous lesions (eg, fibrofolliculomas or trichodiscomas), and increased lifetime risk of renal cell carcinoma (RCC).^20^^,^^21^ FDS affects both sexes equally, with an estimated prevalence of 1.86 per million. Male FLCN carriers exhibit greater pulmonary and cutaneous involvement, while females have a slightly higher renal tumors risk.^22^ Radiologically, FDS is characterized by bilateral pulmonary cysts, predominantly in the lower lobes with peribronchial/subpleural distribution along mediastinal pleura, fissures, and costophrenic sulci (Figure 4). Cysts measure 0.2-8 cm; the largest (>2 cm) are usually lower-lobe, lobulated, and multiseptated. Shapes vary—lentiform, oval, round, lobulated, or irregular—often coexisting within one patient.^23^ Walls are thin yet visible; total cyst count is usually <50, and lesions remain stable or enlarge slowly. Cysts commonly abut or envelop proximal lower-lobe vessels, while intervening lung parenchyma stays normal. SP, arising from ruptured subpleural cysts, affects >60% of FDS patients and recurs frequently, and positive family history aids diagnosis.^24^^,^^25^ Abdominal imaging detects renal neoplasms in up to 27%,^26^ often bilateral, multifocal, and recurrent; subtypes include oncocytoma, chromophobe, or hybrid RCC, whereas clear-cell RCC is uncommon; and metastatic signs should be evaluated.

#### Lymphoid interstitial pneumonia

**Figure 5:**
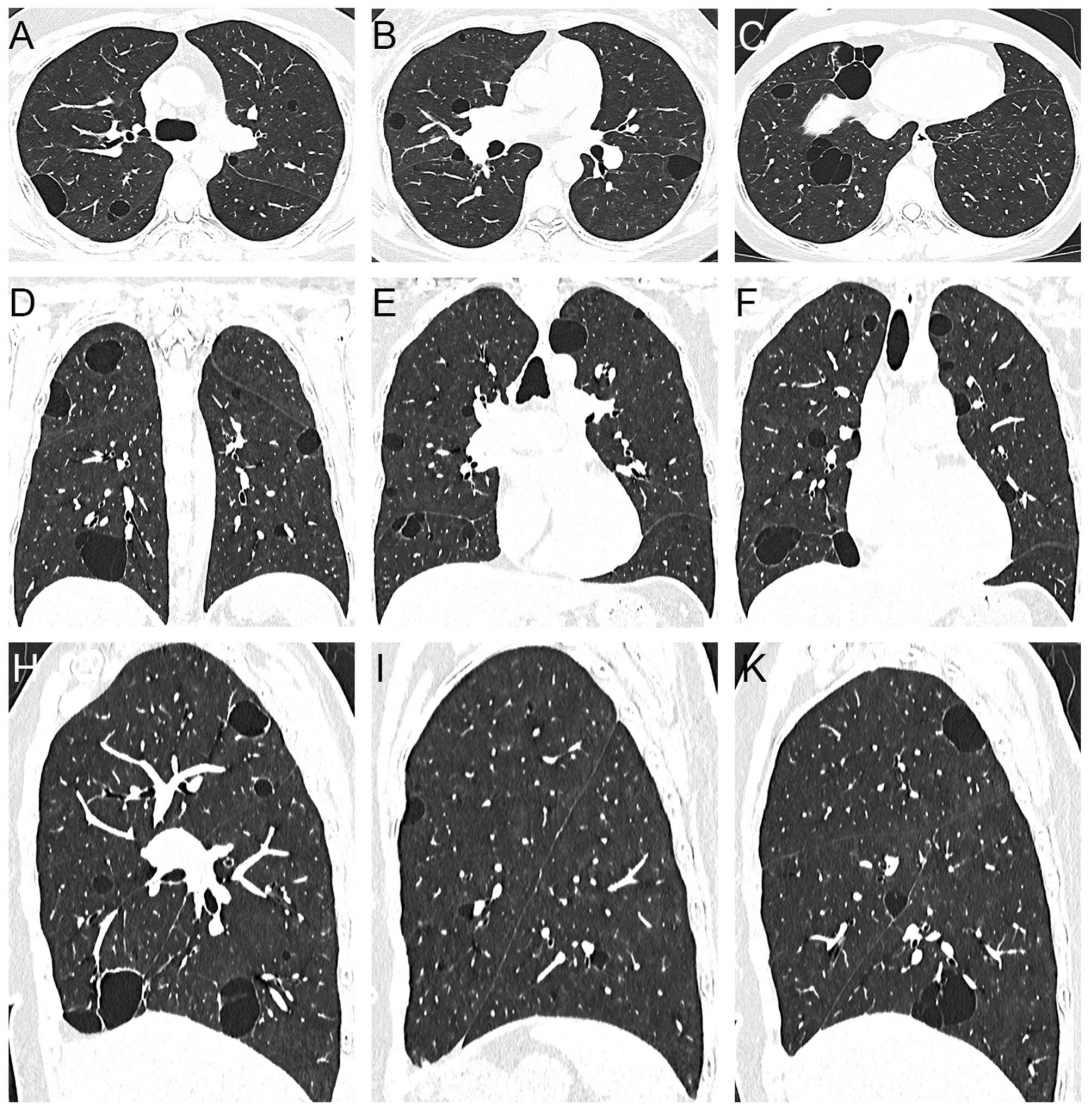
LIP in a 38-year-old woman with Sjögren’s disease. CT images (A-C, axial; D-F, coronal; H-K, sagittal) demonstrate sparse, thin-walled pulmonary cysts of variable shape and size—some septated—clustered in subpleural and peribronchovascular regions of both lungs. Abbreviation: LIP = lymphoid interstitial pneumonia.

Lymphoid interstitial pneumonia (LIP) is a rare interstitial pneumonia representing pulmonary involvement in lymphoproliferative disorders. It predominantly affects women aged 40-60 years and is commonly associated with autoimmune disorders (eg, Sjögren’s disease [SjD], systemic lupus erythematosus, rheumatoid arthritis) or immune-dysregulated states (eg, HIV/AIDS, congenital immunodeficiency, allogeneic hematopoietic stem-cell transplantation). Radiologically, LIP presents with thin-walled cysts—typically sparse (<20), <3 cm, variable in shape, and occasionally septated—diffusely scattered in a bilateral, mid-to-lower lung distribution with subpleural/peribronchovascular predominance and associated GGOs (Figure 5). Cysts lie within or adjacent to GGOs; on follow-up CT, GGOs fade and cysts remain. Additional parenchymal findings include centrilobular and subpleural nodules, thickened interlobular septa and peribronchovascular bundles, bronchiectasis and/or bronchiolectasis. In SS patients, scattered pulmonary cysts strongly suggest LIP, while cavitary nodules signal possible amyloid-associated LIP.^27^ Pneumothorax and pleural effusion are rare, and effusions, especially with large nodules or consolidation, warrant lymphoma evaluation.

#### Amyloidosis

**Figure 6:**
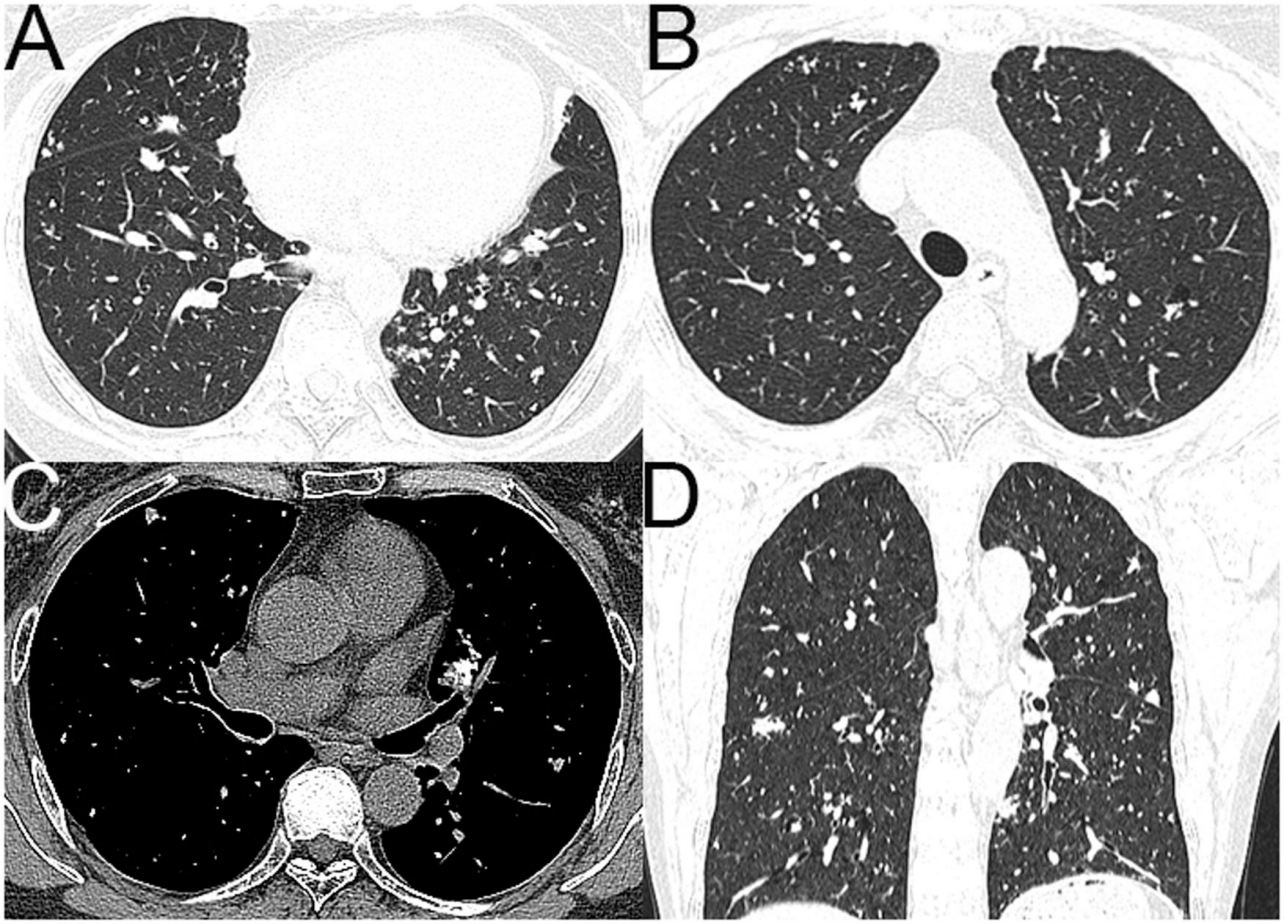
Pulmonary amyloidosis in a 60-year-old woman with Sjögren’s disease. CT images (A-C, axial; D, coronal) demonstrate multiple peribronchial and subpleural nodules in both lungs, varying in size and shape, some calcified. Scattered small cysts are also present, predominantly along peribronchovascular bundles; with mural or intracystic nodules visible within several cysts.

Amyloidosis is characterized by extracellular amyloid deposition, can be systemic or localized.^28^ Four major types are recognized: light chain (AL), amyloid A (AA), amyloid transthyretin (ATTR), and localized amyloidosis. Respiratory involvement occurs in approximately 50% cases,^29^ most often in AL type,^30^ manifesting as tracheobronchial, nodular, diffuse alveolar-septal/parenchymal, or lymphatic forms. A distinct presentation is amyloid-associated cystic lung disease (AACLD), usually linked to localized nodular amyloidosis and associated with collagen-vascular disease or lymphoproliferative disorders, especially SjD or MALT lymphoma.^31^ Radiologically, AACLD typically presents numerous (usually >10), small-to-medium (<2 cm), thin-walled (<2 mm) cysts that are round or lobulated, occasionally septated, and predominantly in the bilateral lower lungs with peribronchovascular and subpleural distribution. These cysts are commonly accompanied by multiple nodules,^31^ often calcified,^32^ variably solid or part-solid, rarely cavitary,^31^^,^^33^ and typically pericystic or intracystic; noncalcified nodules can mimick malignancy.^34^^,^^35^ The coexistence of peribronchovascular/subpleural cysts and calcified nodules (especially when nodules abut cyst walls), particularly in SjD, strongly indicates amyloidosis or lymphoproliferative disease^32^^,^^36^ (Figure 6). Additional image findings include limited GGOs, circumferential airway wall thickening (often calcified), mediastinal/hilar lymphadenopathy (sometimes calcified, more common in systemic/diffuse disease), and pleural effusions or thickening. Over years, asymptomatic patients may show progressive enlargement or increasing numbers of cysts and nodules, accompanied by progressive calcification.^31^

#### Light chain deposition disease

**Figure 7:**
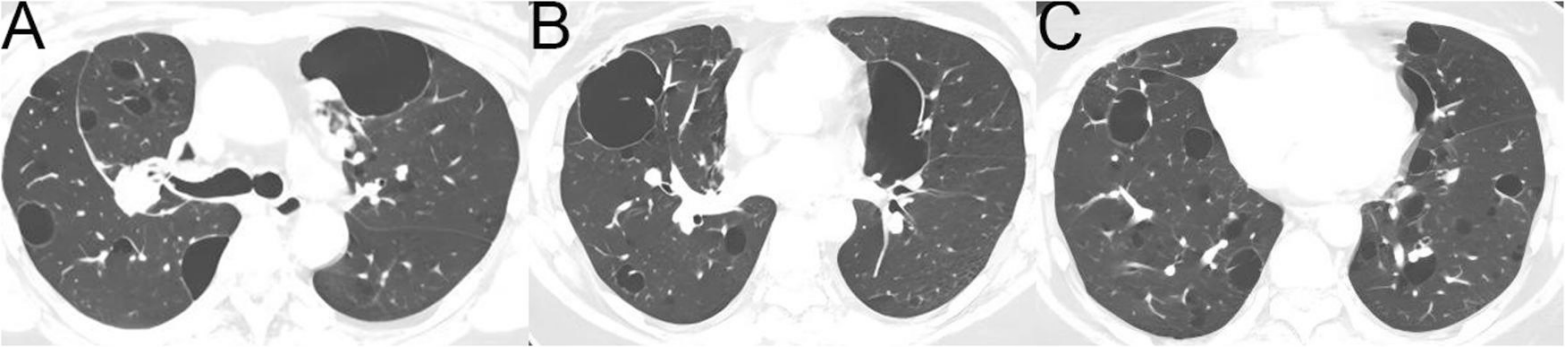
PLCDD in a 46-year-old woman with Sjögren’s disease. CT images (A-C, axial) show multiple bilateral, thin-walled cysts of varying size and shape; intracystic vessels traverse several cyst walls. Abbreviation: PLCDD = pulmonary light chain deposition disease.

Light chain deposition disease (LCDD), a rare systemic disorder, is characterized by non-fibrillar, Congo-red-negative monoclonal immunoglobulin light chains deposition that is distinct from amyloidosis.^37^ Pulmonary LCDD (PLCDD) is uncommon, often asymptomatic, and usually affects middle-aged women in association with lymphoproliferative, plasma-cell, or autoimmune disorders (eg, MALT lymphoma, SjD). Radiologically, PLCDD presents multiple bilateral thin-walled cysts of variable size, often containing intramural or traversing vessels (Figure 7).^36^^,^^38^ Cysts are often diffusely distributed with mild lower-lung predominance; over time, they gradually enlarge, increase in number, eventually coalesce into irregular shapes,^38^ and fuse with varicose bronchiectasis to produce emphysema-like parenchymal destruction.^39^ Additional findings include multiple randomly distributed 2-5 cm solid pulmonary nodules and patchy GGO, suggesting alveolar hemorrhage or superimposed infection.^38^ Bronchial abnormalities—most notably varicoid bronchiectasis—are common; dilated bronchi often communicate with adjacent cysts, compounding structural distortion.^39^ Longitudinal imaging shows both cysts and bronchiectases steadily enlarge and increase in number over time.^40^

#### Castleman disease

**Figure 8:**
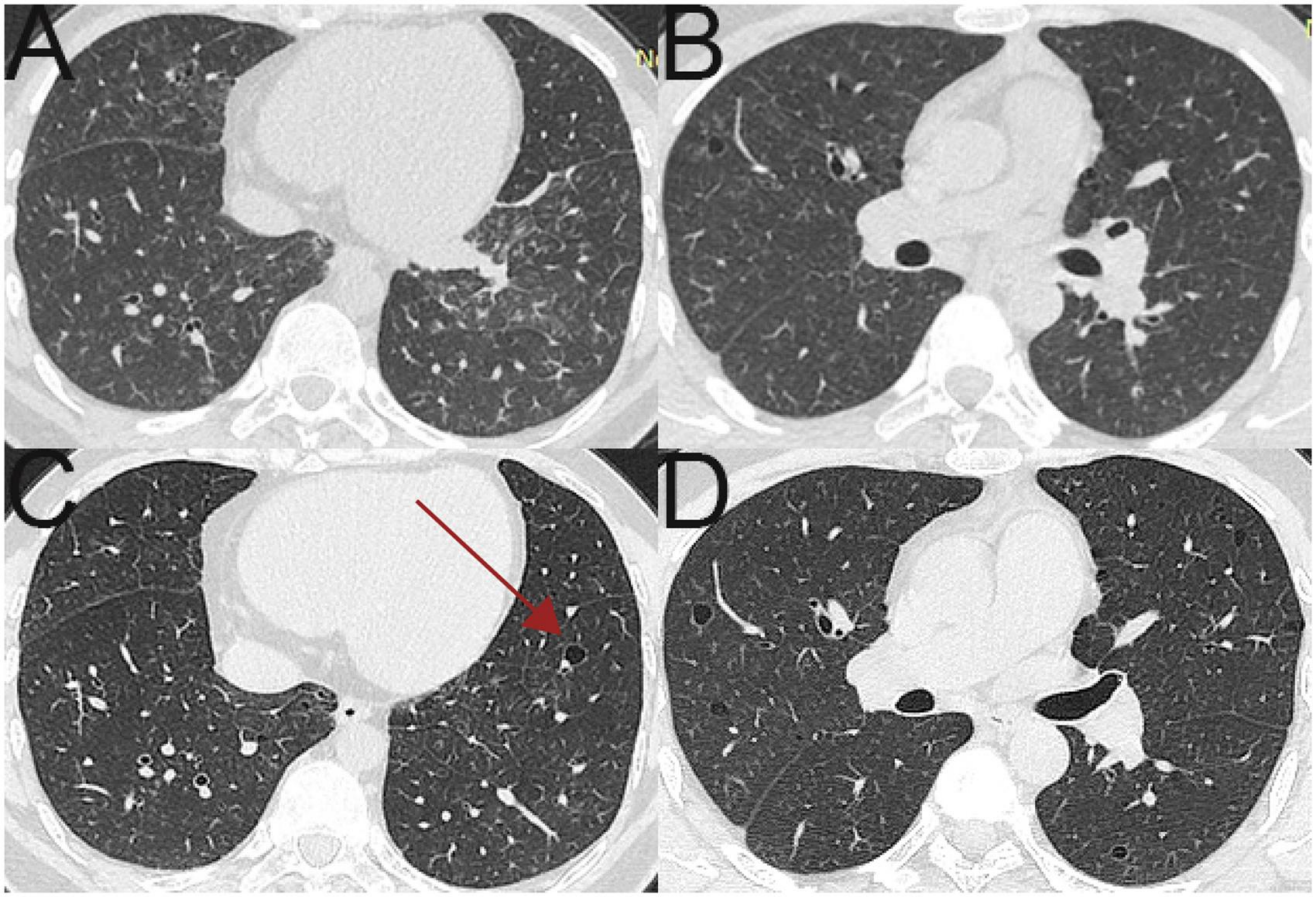
Castleman disease in a 35-year-old woman. Baseline CT images from December 2016 (A-B) show bilateral multiple patchy GGOs and scattered small subpleural/peribronchovascular cysts, mainly in the upper lobes. Follow-up CT in January 2018 (C-D) demonstrates GGOs resolved, with an increased number and size of pulmonary cysts. New cyst appears at the former GGO site in the left lower lobe (red arrow, C). Abbreviation: GGO = ground-glass opacity.

Castleman disease (CD), also termed angiofollicular or giant lymph-node hyperplasia, is a group of rare, heterogeneous lymphoproliferative disorders encompassing unicentric (UCD) and multicentric (MCD) forms. MCD comprises human herpesvirus 8 (HHV-8)-associated MCD (mainly in immunocompromised hosts, especially HIV) and idiopathic MCD (iMCD), which is HIV- and HHV-8-negative.^41^ Radiologically, pulmonary cysts are a hallmark of iMCD, seen in 35%-87% of cases with lung involvement.^42^^,^^43^ The cysts are typically multiple, bilateral, thin-walled, and round-to-oval, though occasional irregular contours with non-smooth walls may occur (Figure 8). Once formed, cysts persist despite therapy.^42^^,^^43^ They raise within or beside GGOs that histologically correspond to plasma-cell infiltration-induced destruction of alveolar elastic fiber, heralding new cyst formation.^43^^,^^44^ Associated nodules are usually numerous, ill-defined, centrilobular, and frequently pericystic or intracystic (mural or central); they may antedate or occasionally evolve into cysts. Sequential imaging shows progression from nodules to cysts and ultimately to consolidation, reflecting chronic parenchymal destruction.^42^ Interstitial changes manifest as thickened bronchovascular bundle and interlobular/intralobular septa. Air-space consolidation and bronchiectasis are uncommon. Extrapulmonary findings include lymphadenopathy, pleural or pericardial thickening/effusion, ascites, and lytic bone lesions.

#### *Pneumocystis jirovecii* pneumonia

**Figure 9:**
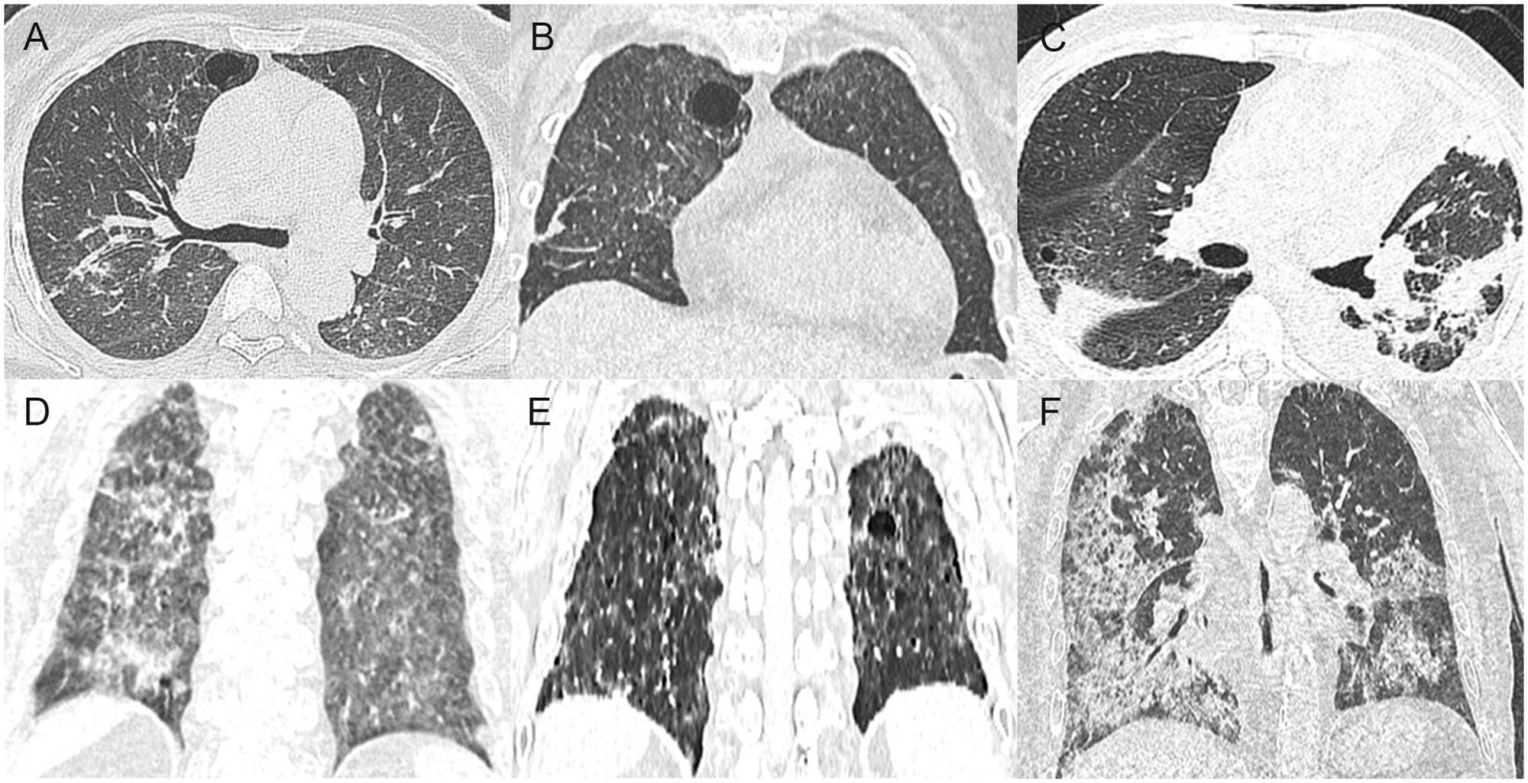
Pulmonary cysts in patients with PJP. CT images (A-E) illustrate that all cysts are located within areas of GGOs. In some cases, the cysts are distributed in a subpleural location (A-B), which may confer a potential risk of cyst rupture and secondary pneumothorax. In other patients, the cysts are surrounded by GGOs with superimposed interlobular and intralobular septal thickening, forming the so-called crazy-paving pattern (C-E, black arrow and arrowhead). Panel F provides a more representative example of the crazy-paving pattern. Abbreviations: PJP = *Pneumocystis jirovecii* pneumonia; GGO = ground-glass opacity.

*Pneumocystis jirovecii* pneumonia (PJP, formerly called *Pneumocystis carinii* pneumonia or PCP) is a life-threatening opportunistic fungal infection predominantly affecting immunocompromised hosts. Radiologically, pulmonary cysts are reported in up to 34% of PJP patients in selected cohorts.^45^ The cysts are typically multiple, bilateral, thin-walled, and 1-5 cm in size, with an upper-lobe predominance, particularly in patients receiving aerosolized pentamidine prophylaxis. They usually arise within areas of preexisting ground-glass opacities and are associated with a high risk of rupture, resulting in SP, pneumomediastinum, or subcutaneous emphysema (Figure 9). Although cysts often regress after effective treatment, newly formed cysts during therapy may indicate persistent vulnerability to air-leak complications. Compared with HIV-associated PJP, non-HIV immunocompromised patients more frequently show extensive GGOs, consolidation, crazy paving, and centrilobular nodules, whereas cyst formation is less common; however, once cysts are present, they carry the same risk of air-leak complications. Advanced or progressive disease may demonstrate bronchiectasis, fibrosis, and architectural distortion, while pleural effusions and mediastinal lymphadenopathy remain uncommon and should raise suspicion for alternative or concomitant diagnoses.

#### Alveolar macrophage pneumonia

**Figure 10:**
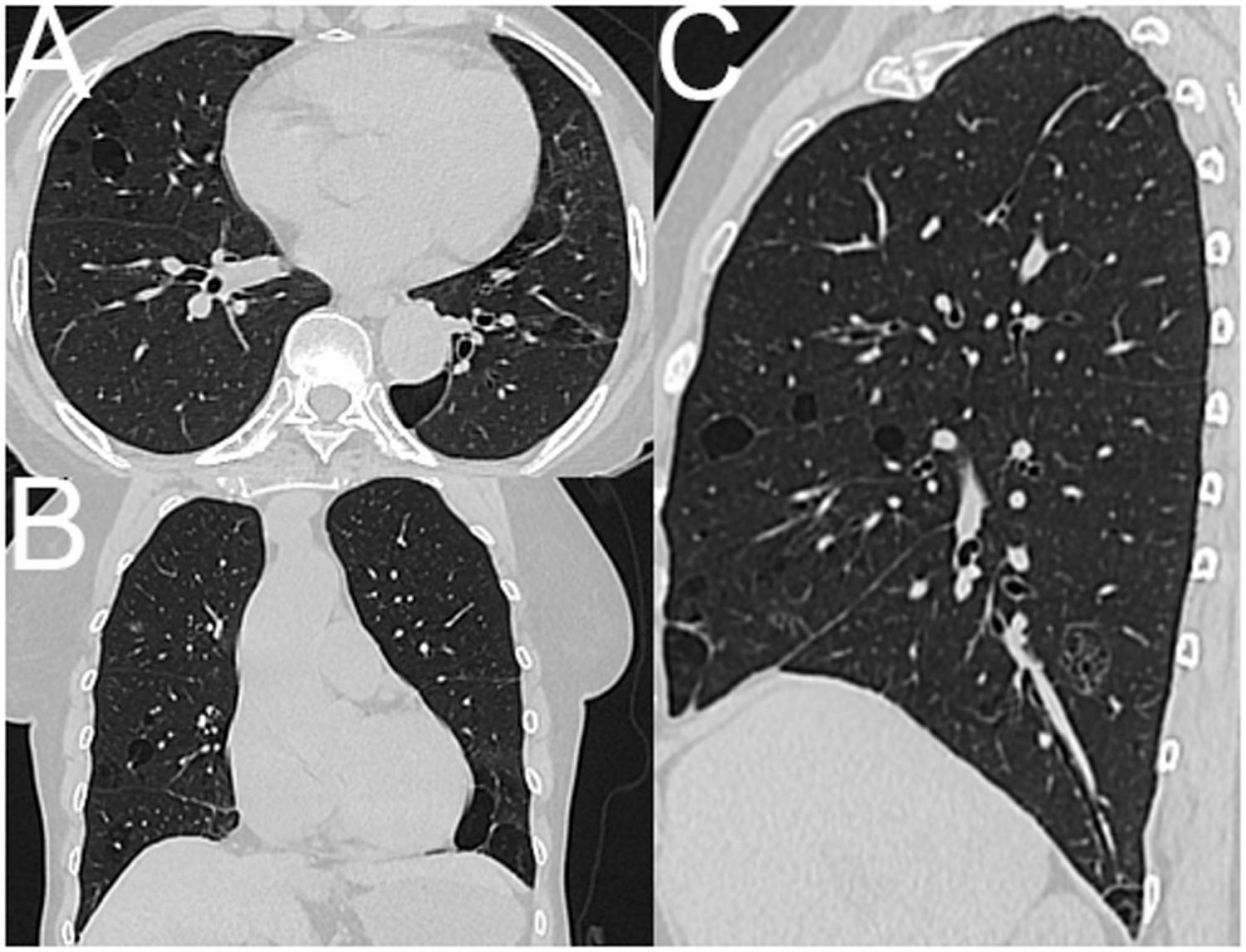
AMP in a 67-year-old nonsmoking woman with systemic lupus erythematosus. CT images (A-C) show multiple thin-walled cysts of variable size in the right middle lobe and both lower lobes, predominantly subpleural. Abbreviation: AMP = alveolar macrophage pneumonia.

Alveolar macrophage pneumonia (AMP, formerly called desquamative interstitial pneumonia) is a rare idiopathic interstitial pneumonia that mainly affects middle-aged adults (30-60 years) and is strongly associated with heavy smoking; it presents with progressive dyspnea and dry cough.^46^ Non-tobacco-related cases occur rarely in the setting of autoimmune disorders, occupational or environmental exposures, or drug toxicity.^47^ AMP is radiologically characterized by cysts and GGOs. In 30%-75% of patients, small (usually <2 cm), well-defined, thin-walled cysts, often uniform in shape and delicate in wall, are embedded within GGOs. They predominantly involve the lower-lobe periphery, usually occupy <10% of parenchyma, and may be discrete or clustered (Figure 10). GGOs are predominantly bilateral (80%) and basal (90%), often symmetric and peripheral (60%), patchy (20%), or diffuse (18%), occasionally accompanied by reticular or linear opacities and mild traction bronchiectasis.^47^ Unlike other fibrosing pneumonias, AMP shows minimal architectural distortion; honeycombing, if present, is peripheral and limited to <1/3 of cases, and short-term progression to UIP is uncommon.^47^ Complications include pneumothorax and hemopneumothorax.^48^ AMP generally responds well to treatment, especially smoking cessation, resulting in regression of GGOs and cystic changes on follow-up CT.

## Imaging diagnostic steps of common DCLDs

CT, particularly HRCT, is the cornerstone for evaluating DCLDs, precisely delineating cyst morphology and associated findings. Because HRCT patterns are frequently nonspecific, they must be interpreted within the clinical context to guide further work-up. A systematic protocol is recommended: first survey intrapulmonary findings, then extrapulmonary, and extrathoracic manifestations, and finally refine the diagnosis using characteristic, stepwise features.

### Key CT observations for cystic lung diseases

1. Intrapulmonary manifestations: first, confirm true cysts and characterize the location, distribution, number, size, shape, margin, internal structures, and wall conditions of the cyst. Second, assess accompanying findings, including nodules, GGOs, other parenchymal abnormalities, and the intervening lung parenchyma, with particular attention to their temporal and spatial relationships with cysts.
2. Extrapulmonary thoracic manifestations: assess the pleura for thickening, calcification, or masses; screen for pneumothorax or pleural effusion; examine the mediastinum for cyst lesions, lymphadenopathy, or pneumomediastinum; and survey the chest wall for bone or soft-tissue abnormalities.
3. Extrathoracic manifestations: neck: scan for thyroid lesions (likely supplementary findings better characterized on other imaging modalities)^49^ or subcutaneous emphysema^50^; abdomen/pelvis: identify organ-based abnormalities that may clarify etiology; these areas are often included on chest CT. Optimize window width/level settings and review the scout image for additional clues. When imaging or clinical data are insufficient for a definitive diagnosis, promptly communicate with the referring clinician or recommend further targeted evaluation.
4. Diagnostic integration: correlate HRCT findings with clinical and laboratory data; supplement with targeted multimodal imaging when necessary, and communicate with clinicians when imaging alone is inconclusive.

### Diagnostic steps for DCLDs based on CT

#### Initial assessment: confirming cystic lesions

**Figure 11:**
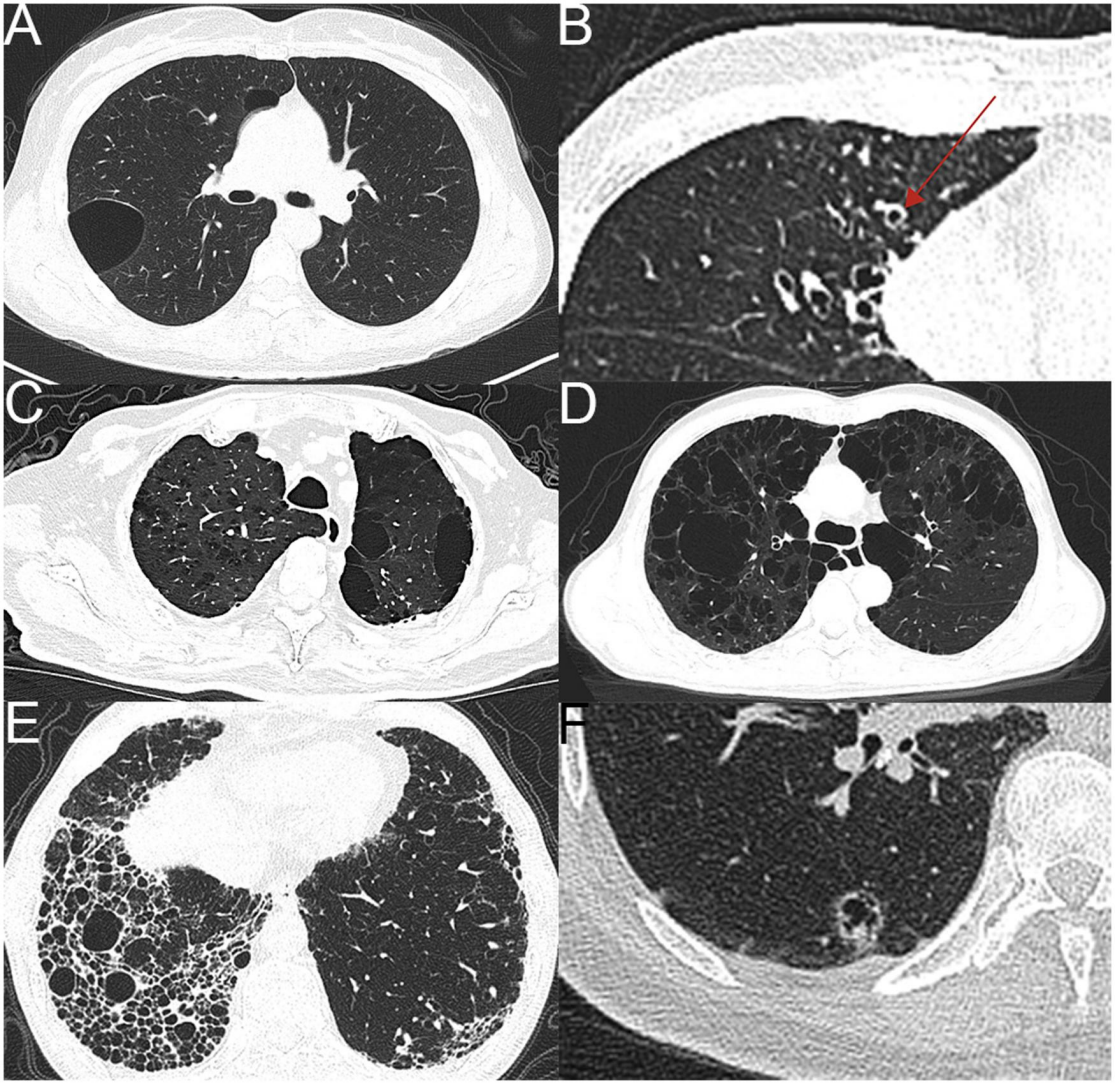
Common cystic and cyst-like lesions. Bulla (A): large (>1 cm), air-containing structures with very thin walls. Cystic bronchiectasis (B): dilated bronchi manifest as non-tapering tubular structures extending peripherally to within 1 cm of the pleura; their continuity across adjacent axial and coronal planes helps differentiate them from isolated cysts. The “signet ring” sign (red arrow) is evident, with a dilated bronchus appearing as a ring adjacent to a smaller pulmonary artery that mimics the signet of a ring. Emphysema (C-D): centrilobular and paraseptal emphysema (C); panlobular emphysema (D). Honeycombing (E): bilateral, predominantly peripheral, and basal-predominant honeycombing is observed, characterized by clustered cystic air spaces of variable size with well-defined walls. Cystic lung cancer (F): a multilobulated cystic lesion measuring approximately 1.5 cm in the right lower lobe. The lesion shows irregular wall thickening with indistinct margins and contains an eccentric mural nodule and internal septations.

The initial step in DCLD evaluation is to distinguish true cysts from common mimics (Table 2; Figure 11) by anatomical location (parenchymal vs subpleural) and distribution. Once confirmed, cyst characterization, including number, distribution, shape, internal features, and wall thickness, guides differentiation diagnosis. Accurate cyst enumeration is essential. A solitary pulmonary cyst typically represents an incidental finding, such as an age-related cyst or pneumatocele, and rarely warrants further evaluation. Isolated or sporadic cysts are frequently observed in asymptomatic adults over 40 years old, often appearing as solitary, lower-lobe-predominant lesions that remain stable over time and are not associated with smoking or significant functional impairment.^55^ In contrast, DCLDs typically present with multiple cysts (≥5), characteristic distributions, and associated imaging findings, necessitating further diagnostic assessment. In DCLDs, cyst distribution and morphology are the most discriminating CT features. Diffuse, round parenchymal cysts are characteristic for LAM; bizarre-shaped, upper- and mid-lung cysts indicate PLCH; and irregular, basal-subpleural cysts point to FDS. Internal cystic features provide additional diagnostic refinement. Cysts in pulmonary amyloidosis frequently harbor mural or central nodules; those in LIP often contain internal septations; and cysts associated with LCDD characteristically demonstrate traversing vessels. Conversely, cysts in LAM are typically devoid of septations, vessels or other internal features. Wall thickening is inherently variable and may fluctuate secondary to concomitant infection, lung parenchymal compression, or other local factors, thereby necessitating cautious interpretation. Table 3 provides a concise, side-by-side comparison of the key cystic imaging characteristics observed in the major DCLDs in which cysts constitute the dominant radiologic manifestation.

**Table 2:** Differential features of true cysts versus common pulmonary cyst mimics.

Cystic lesion	Pathology/anatomy	Radiology	Remarks	References
Bleb	Small air-containing space (<1 cm), located within the visceral pleura or subpleural lung	Thin-walled, low-attenuation lucency adjacent to the pleura	Associated with small-airway disease and pneumothorax, especially in active smokers; distinguishing from a bulla is arbitrary and discouraged	51
Bulla	An airspace >1 cm with thin wall (<1 mm) composed of a thin layer of collapsed lung tissue, resulting from alveolar destruction	Round, low-attenuation lucency with an imperceptible wall, typically subpleural and most prominent in the lung apices (Figure 10A)	Associated with pulmonary centrilobular and paraseptal emphysema, resulting from dilatation, destruction, and confluence of airspaces distal to terminal bronchioles	51
Cyst	Dilated spaces within the tissue lined by epithelial cells or fibrous structures	Round parenchymal lucencies or low-attenuating areas with well-demarcated interfaces against adjacent normal lung tissue	Usually thin (<2 mm) and regular walls, predominantly air-filled lumina, though may occasionally contain fluid or solid components	51
Cavity	Air- or fluid-filled space with thick, often irregular wall, resulting from necrosis and drainage	Focal lucency or low-attenuation area with thick wall (>4 mm), often associated with consolidation or mass	Differentiate from cyst (wall <2 mm). May contain air-fluid level. Pseudocavities represent spared parenchyma, normal or ectatic bronchi, or emphysema	52
Cystic bronchiectasis	Severely dilated bronchi with cystic morphology extending to pleural surfaces	Clustered, round lucencies with visible airway walls (“bunch of grapes”, “tram-track”, “signet ring”) near pleura (Figure 10B)	Caused by bronchial wall destruction, often accompanied by infection, peribronchial fibrosis, and scarring	53
Emphysema	Irreversible airspace enlargement distal to terminal bronchioles, with alveolar wall destruction. Three major subtypes: centrilobular, panlobular, and paraseptal	Lucencies without visible wall, often with central vessels (Figure 10C and D)	May coexist with local fibrosis. Air in the interstitium (“interstitial emphysema”) or soft tissues (“subcutaneous emphysema”) from barotrauma; not related to parenchymal destruction	51
Honeycombing	Stacked cystic spaces resulting from fibrotic collapse of alveoli and dilation of alveolar ducts and lumens, with subpleural distribution	Subpleural clustered cysts (3-10 mm) with 1-3 mm walls, associated with architectural distortion and often coexisting with traction bronchiolectasis (Figure 10E)	Indicates end-stage fibrosis. Use only when supporting fibrotic features are present. May be present microscopically without CT correlation.	51
Cystic lung cancer	Typically adenocarcinoma, with rare cases of adenosquamous carcinoma, neuroendocrine tumors, and lymphomas; cystic change may be due to check-valve mechanism	Cystic nodule with solid components (exophytic or endophytic), irregular wall thickening, septations, ground-glass opacity, or multilocular space (Figure 10F)	Suggestive features: irregular wall, eccentric solid parts, multilocular spaces. Solidification or cavitation may occur; should be assessed on serial CT	54
Pneumatocele	Pseudocyst; gas-filled, thin-walled cystic spaces without epithelial lining or bronchial wall elements	Thin-walled, smooth, gas-filled cyst, sometimes with air-fluid level. Often transient	Common post-infectious, traumatic, or related to mechanical ventilation	6

**Table 3:** Imaging hallmarks of cyst-predominant diffuse cystic lung diseases.

Feature	LAM	FDS	PLCH	LIP
Cyst shape	Uniform round or oval	Variable (lentiform, oval, or lobulated)	Bizarre shapes (bilobed, cloverleaf, branched)	Variable (round to irregular)
Cyst distribution	Diffuse symmetric distribution; costophrenic angles involved	Basal and mediastinal; peribronchial and subpleural	Upper or mid- lung; costophrenic angles and lung bases spared	Bilateral mid and lower lung; subpleural and peribronchovascular
Cyst size	Mild to moderate (≤20 mm)	Moderate to marked (≤80 mm)	Mild to moderate (≤20 mm)	Mild to moderate (≤30 mm)
Cyst internal structures				
Vessels	Absent	May be present	Absent	Absent
Septations	Absent	May be present in large cysts	Absent	May be present
Cyst wall				
Thickness	Thin	Thin	Thick or thin	Thin
Wall-associated components	Absent	Absent	Nodule may be present	Absent
Cyst burden	Profuse	Moderate	Moderate	Sparse

Abbreviations: FDS = folliculin deficiency syndrome; LAM = lymphangioleiomyomatosis; LIP = lymphoid interstitial pneumonia; PLCH = pulmonary Langerhans cell histiocytosis.

#### Further evaluation: integrating ancillary imaging features

Beyond cyst morphology, a systematic appraisal of ancillary radiologic findings, including the distribution of pulmonary nodules, the presence and pattern of GGOs, the integrity of intervening lung parenchyma, and both intra- and extrathoracic manifestations, substantially refines the differential diagnosis of DCLDs. Multiple upper-lobe nodules with possible cavitation (“cheerio sign”) suggest PLCH, whereas poorly defined centrilobular or subpleural nodules interspersed with cysts favor LIP, particularly in patients with autoimmune diseases such as SS. The presence of solid or subsolid nodules in a patient with cystic lung disease may suggest TSC-LAM, but requires integration with the diagnostic criteria outlined above for confirmation. A concomitant predominance of cysts and GGOs typifies LIP and is frequently accompanied by interlobular septal thickening and additional micronodules. By contrast, other DCLDs generally lack GGOs; nevertheless, PLCH complicated by smoking-related interstitial lung diseases (ILDs), namely respiratory bronchiolitis or AMP, may exhibit such opacities. Non-lesional parenchyma appears radiologically normal in PLCH and FDS, whereas background smoking-related changes support PLCH. Intrathoracic extrapulmonary findings provide further discriminatory value: SP occurs in LAM, FDS, and PLCH; pleural effusion is most prevalent in LAM; and mediastinal lymphadenopathy raises suspicion for sarcoidosis or lymphoma. Finally, extrathoracic manifestations, including renal AMLs in LAM, neurological findings in TSC, renal neoplasms in FDS, osseous lesions in PLCH/CD, and autoimmune or immunodeficiency markers in LIP, serve as critical adjuncts for definitive diagnosis. For more details, please refer to the preceding section detailing the imaging features of DCLDs.

#### Comprehensive assessment: integrating clinical context

**Figure 12:**
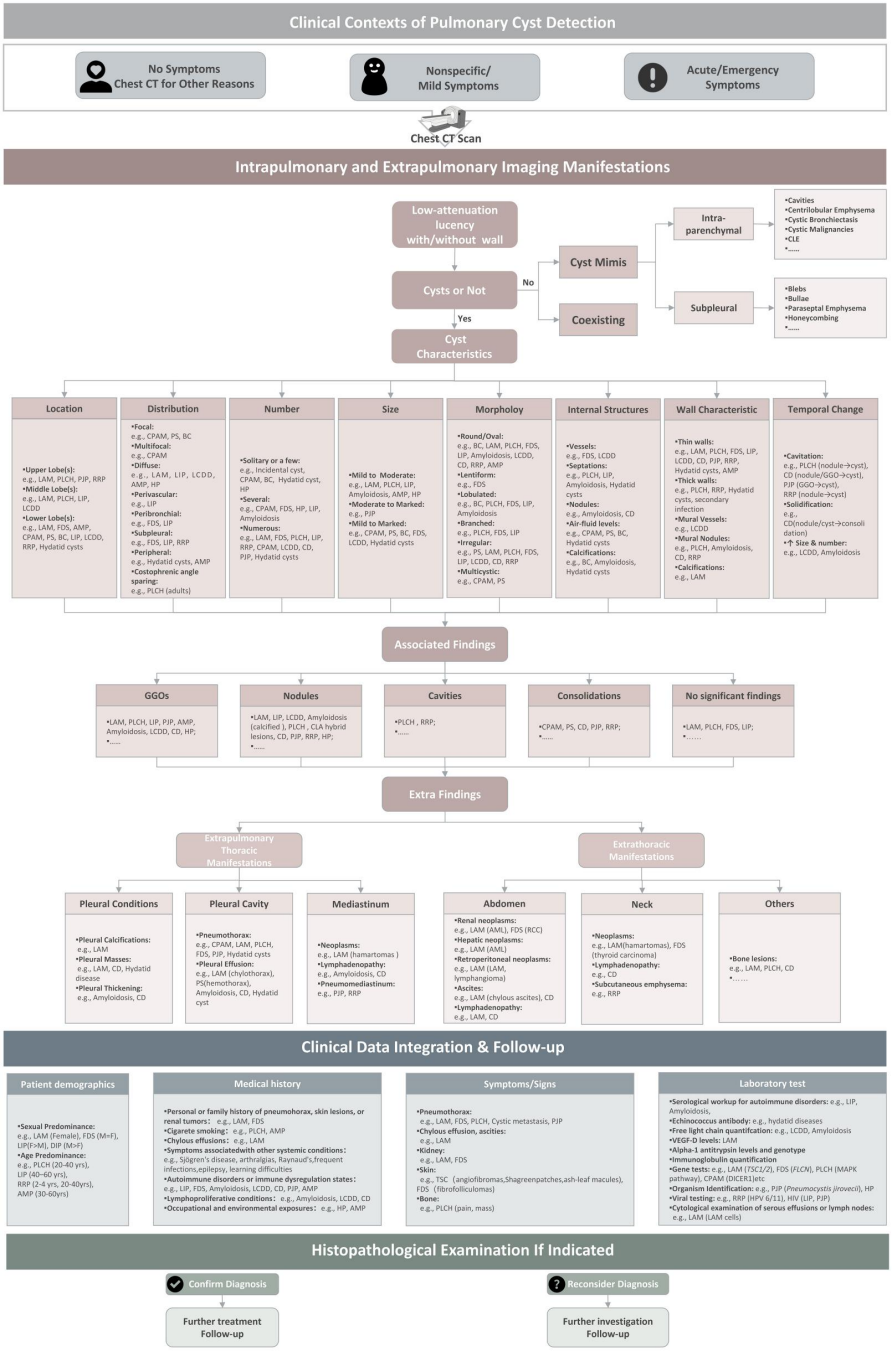
A hierarchical diagnostic approach for cystic lung diseases based on CT. Cyst size was classified as *mild* (≤20 mm), *moderate* (20-80 mm), or *marked* (>80 mm). Abbreviations: AML = angiomyolipoma; AMP = alveolar macrophage pneumonia; BC = bronchogenic cyst; BMD = bone mineral density; CD = Castleman disease; CLE = congenital lobar emphysema; CLM = congenital lung malformations; CPAM = congenital pulmonary airway malformation; FDS = folliculin deficiency syndrome; GGO = ground-glass opacity; HP = hypersensitivity pneumonitis; LAM = lymphangioleiomyomatosis; LCDD = light chain deposition disease; LIP = lymphoid interstitial pneumonia; PJP = *Pneumocystis jirovecii* pneumonia; PLCH = pulmonary Langerhans cell histiocytosis; PS = pulmonary sequestration; RRP = recurrent respiratory papillomatosis; TSC = tuberous sclerosis complex; VEGF = vascular endothelial growth factor.

Accurate diagnosis of DCLDs requires a multidisciplinary synthesis of HRCT findings with detailed clinical data, ancillary investigations, and, when necessary, targeted histopathology. Although imaging often provides the initial diagnostic lead, interpretation must be contextualized by age, sex, reproductive status, lifetime smoking exposure, familial clustering, systemic comorbidities, current medications, extrathoracic manifestations, and relevant environmental or occupational exposures. For example, recurrent SP in a young, nonsmoking woman with renal AMLs is diagnostic for LAM, which aligns with the diagnostic criteria requiring HRCT plus an extrapulmonary manifestation, whereas familial pneumothorax in conjunction with fibrofolliculomas in a middle-aged adult points to FDS. Progressive exertional dyspnea accompanied by upper-lobe cysts and centrilobular nodules in a young smoker indicates PLCH; conversely, a similar imaging constellation with superimposed GGOs in a middle-aged smoker may indicate AMP. Smoking history is pivotal: PLCH is rare in never-smokers, whereas emphysematous bullae, common in smokers, may simulate cysts yet lack discrete walls. Targeted genetic testing further refines hereditary DCLDs, including *FLCN* mutations in FDS, *TSC1/TSC2* mutations in LAM, and *FBN1* variants in Marfan syndrome. Beyond primary syndromes, environmental/occupational exposures offer crucial diagnostic clues. Chronic hypersensitivity pneumonitis (HP), triggered by repeated inhalation of organic/inorganic antigens, may culminate in cystic remodeling in advanced fibrotic stages, particularly in nonsmoking patients. Laboratory biomarkers complement clinical reasoning: in a young woman with diffuse thin-walled cysts and a confirmed history of TSC, the diagnosis strongly favors LAM. In the absence of TSC, elevated VEGF-D levels support sporadic LAM, whereas normal values heighten suspicion for FDS or PLCH, although LAM cannot be definitively excluded. Dermatologic or rheumatologic evaluations may resolve ambiguous imaging scenarios by revealing cutaneous stigmata of TSC or autoimmune markers of LIP. When diagnostic uncertainty persists and accurate classification directly influences prognostication or therapy, video-assisted thoracoscopic or transbronchial biopsy is warranted. Guided by these considerations, we propose a hierarchical diagnostic framework for DCLDs (Figure 12).

## Comprehensive imaging in the diagnosis, management, and follow-up of DCLDs

Disease trajectories in DCLDs are variable; imaging that captures both complications and evolution is therefore essential for patient management.

### Early detection and diagnosis

Early recognition of DCLDs prevents respiratory failure, pneumothorax, and malignant transformation. HRCT-based screening should target high-risk populations: individuals with pathogenic germline variants (eg, FLCN, TSC1/2), recurrent SP, or unexplained dyspnea in young nonsmokers. SP may be the first manifestation of DCLDs (∼10% of apparent primary cases),^56^ often at a younger age.^57^ Although guidelines do not mandate CT after a first episode of SP, cost-effectiveness analyses favor HRCT screening for early detection and recurrence reduction. Current international TSC recommendations advise chest CT for women ≥18 years and symptomatic men with TSC to screen for LAM.^58^ When biopsy is contraindicated, single-system PLCH can be diagnosed in smokers by typical CT findings and exclusion of other etiologies.^59^ Radiologists must advocate targeted screening while minimizing overdiagnosis.

### Monitoring disease progression

Serial CT quantifies cyst volume, number, and distribution, correlating with symptom development and long-term functional decline in DCLDs. In LAM, progressive cyst enlargement predicts accelerated forced expiratory volume in the first second (FEV_1_) decline and guides initiation of mammalian target of rapamycin (mTOR) inhibitor therapy,^60^^,^^61^ whereas numerous ultra-small cysts may impair gas exchange despite preserved conventional spirometry.^62^ In FDS, higher cyst burden, particularly subpleural and lower-lobe regions, heightens pneumothorax risk despite preserved ventilation function.^63^ In CLMs, lesions occupying >18% of lung volume or exhibiting hyperdensity forecast symptomatic deterioration.^64^ Integrating quantitative imaging with pulmonary function and biomarkers permits precision surveillance.

### Assessing response to treatment

Therapeutic efficacy is evaluated by multimodal assessment. In LAM, serial CT enables quantitative assessment of sirolimus-mediated stabilization or regression of cyst volume and air trapping.^65^^,^^66^ PLCH may show nodule regression and halted cyst expansion after smoking cessation and cladribine therapy.^67^^,^^68^ Infectious cysts resolve as cyst wall thickness and consolidation decrease after treatment.^69^ Quantitative imaging parameters, including total cyst volume, cyst number, and wall characteristics, are critical in monitoring response. Functional and metabolic imaging modalities complement anatomical assessment by tracking ventilation improvements and metabolic activity during treatment course.^70^ However, challenges persist, including pseudoprogression, radiation burden from repeated CT scans, and heterogeneous responses in multifocal diseases.

### Follow-up protocols and management

Evidence-based follow-up schedules for common DCLDs remain heterogeneous (Table 4). In practice, frequency and duration are individualized by disease entity, patient risk factors, and clinical context, balancing early detection against radiation exposure and resource utilization. Prospective studies are needed to standardize disease-specific protocols, including criteria for imaging cessation. A synoptic overview from screening to treatment is summarized in Table 5.

**Table 4:** Imaging follow-up and screening protocols for common diffuse cystic lung diseases.

Disease	Target organ/system	Recommended imaging follow-up	Notes/considerations	Key references
CPAM/CLM	Fetal/neonatal lung	Antenatal: Asymptomatic: serial prenatal ultrasound Symptomatic: fetal MRI Postnatal: All prenatally diagnosed: CXR at birth Asymptomatic + low risk: advanced CT/MRI within 6 months; then annual CXR or CT Asymptomatic + high risk: immediate advanced CT or MRI Symptomatic at birth: immediate CT or MRI Incidental finding postnatally: CT or MRI as needed Symptomatic postnatally: CXR, then CT/MRI if indicated	High-risk indicators include large lesions on CXR, bilateral/multifocal cysts, DICER1 mutations, family history of DICER1 syndrome or PPB-associated conditions	3, 71–73
LAM	Lung, brain, abdomen, bone	Lung: Serial HRCT for suspected LAM TSC women: screen at 18, repeat at 30-40 if negative, or sooner if symptomatic TSC men: HRCT if symptomatic PA: echo for PA pressure in severe cases, long-term oxygen users, or pre-transplant evaluation Brain: MRI for meningioma if symptomatic or on/planned progestins Abdomen: Asymptomatic renal AML <4 cm: annual ultrasound (US); CT/MRI if US unreliable Renal AML ≥4 cm or aneurysm ≥5 mm: biannual US; consider intervention Bone: BMD screening in postmenopausal women	Tailor follow-up by TSC status, symptoms, PFTs, and treatment course	3, 13
TSC-associated LAM	Lung, brain, kidney, heart	Lung: Asymptomatic adult females (negative CT): CT every 5-7 years until menopause LAM findings on CT: follow-up CT as per case (symptoms, PFTs, mTORi, response, comorbidities) Brain: MRI every 1-3 years if <25; more often if SEGA enlarges or hydrocephalus Kidney: MRI every 1-3 years lifelong Heart: Asymptomatic children: echo every 1-3 years until rhabdomyoma regression Symptomatic patients: more frequent or advanced cardiac evaluation	Continue SEGA imaging into adulthood; tailor imaging by symptoms and tumor burden	58
FDS	Lung, kidney, others	Lung: baseline low-dose HRCT ≥20 years old; repeat only if needed Kidney: MRI every 1-2 years lifelong from age 20, More frequent if tumor present Others: no routine imaging for thyroid/salivary/skin/colorectal tumors	Surgical intervention for renal tumors ≥3 cm; PRDM10-related tumors may not follow 3 cm rule. Evaluate post-ablation imaging changes	74, 75
PLCH	Lung, bone, pituitary, multisystem	Initial: whole-body PET/CT Lung (single-system): HRCT at 3-6 months, then 6-12 months; CXR if mild Bone: PET/CT every 2-3 months, then every 3-6 months Pituitary: endocrine evaluation at 3 months, then annually; MRI if initially involved Multisystem: PET/CT every 3-6 months, then every 3-6 months	Organ-specific imaging (CT/MRI) as per involvement Individualize frequency after disease stabilization. Use RECIST/PERCIST to assess response	59

Abbreviations: AML = angiomyolipoma; BMD = bone mineral density; CLM = congenital lung malformations; CPAM = congenital pulmonary airway malformation; CXR = chest X-ray; Echo = echocardiography; FDS = folliculin deficiency syndrome; HRCT = high-resolution CT; LAM = lymphangioleiomyomatosis; PA = pulmonary artery; PERCIST = positron emission tomography response criteria in solid tumors; PET = positron emission tomography; PH = pulmonary hypertension; PLCH = pulmonary Langerhans cell histiocytosis; RECIST = response evaluation criteria in solid tumors; SEGA = subependymal giant cell astrocytoma; TSC = tuberous sclerosis complex.

**Table 5:** Synopsis of key diffuse cystic lung diseases.

Disease		LAM	FDS	PLCH	LIP	PJP
Demographics/associated conditions		Female predominance (especially of childbearing age); may be sporadic or associated with TSC	No sex predominance; positive family history of pneumothorax, skin lesions and/or renal tumors; *FLCN* mutation; autosomal dominant disorder	No sex predominance; young/Middle-aged adults; cigarette smoking; BRAF and other MAPK pathway mutations (eg, ARAF, NRAS, KRAS, MAP2K1, MAP3K1); tumors (Hodgkin’s lymphoma)	Female predominance; females 40-60 years; autoimmune disorders and immune dysregulation (eg, Sjögren’s syndrome, RA, SLE, CVID, AIDS)	Immunosuppressed patients (eg, AIDS, hematological malignancies, organ transplants, prolonged immunosuppressive therapy)
Clinical manifestations	Pulmonary manifestations	Progressive dyspnea; recurrent pneumothorax, chylothorax	Spontaneous pneumothorax (often recurrent)	Cough, dyspnea, chest pain, pneumothorax	Cough, dyspnea, less commonly pneumothorax	Fever, non-productive cough, chest discomfort (worsening chest pain), dyspnoea (particularly on physical exertion), and hemoptysis (rare)
	Extrapulmonary manifestations	Renal AMLs; chylous effusions (chylothorax, chylous ascites); retroperitoneal LAM; abdominopelvic lymphadenopathy TSC-LAM: CNS (SEGAs, cortical tubers, cognitive impairment, epilepsy); skin (Shagreen patches, ash leaf lesions, facial angiofibromas, subungual fibromas); eye (retinal phakomas); hepatic and renal AMLs	Kidney: renal tumors (mixed eosinophilic-chromophobe tumor, chromophobe carcinoma, oncocytoma, and renal AMLs); skin: fibrofolliculomas, trichodiscomas, ± acrochordons	Bone: lytic bone lesions (incidental finding or produce localized pain or a pathologic bone fracture; often involving flat bones); CNS: diabetes insipidus; skin: brown to purplish papules and eczematoid or seborrhea-like lesions	Features of associated underlying conditions (eg, sicca syndrome, arthralgias, Raynaud, history of infections)	Weight loss and chills
Imaging findings	Features of the cysts	TSC-LAM: diffuse nodular lesions along with thin-walled cysts, especially when MMPH coexists S-LAM: thin-walled cysts surrounded by normal parenchyma Homogeneous, diffuse with no geographic predilection	Variable size, round to elliptical or lentiform in shape, thin-walled, air-cuffing. Peripheral, basal-predominant, perivascular, subpleural, and adjacent to mediastinum	Variable size, bizarre shapes, variable wall-thickness, associated with nodules and thick-walled cavities. Superior lobes (upper and middle predominant), spares costophrenic angles, reticulonodular changes in late stages	Variable size (<3 cm), smooth, Variable shapes (eg, round, oval, and irregular), thin-walled, often have eccentric vessels and internal septations. Random, subpleural, basilar, perivascular. Diffuse with slight lower-lobe predominance	Variable size, wall-thickness and shape. Superior lobes
	Other thoracic findings	GGO, septal thickening, lymphadenopathy, pleural effusion, pneumothorax	Pneumothorax	Centrilobular and peribronchial nodules, cavitating nodules, lymphadenopathy, pneumothorax, and lung carcinoma	GGO, centrilobular and subpleural nodules, septal thickening, and lymphadenopathy	GGO, consolidations, septal thickening, and pneumothorax
	Extrathoracic findings	Renal AMLs; chylous effusions (chylothorax, chylous ascites); retroperitoneal LAM; abdominopelvic lymphadenopathy; uterine fibroids-TSC (eg, tubers)	Cutaneous fibrofolliculomas; renal tumors	Only in systemic LCH (lytic lesions in flat bones, etc)	Features of associated underlying conditions such as sicca, arthralgias, Raynaud, history of infections	Extra-pulmonary Pneumocystis jiroveci infection (eg, CNS, liver, spleen, gallbladder, pancreas, bone marrow, lymph node, eye, gastrointestinal tract, and thyroid)
Diagnosis		Characteristic imaging features plus 1 of the following: TSC, renal AML, chylous effusions, lymphangiomyoma, elevated VEGF-D ≥ 800 pg/mL, cytological detection of LAM cells or clusters in serous effusion or lymph nodes, histopathological confirmation of LAM in lung, retroperitoneal, or pelvic tumors; lung biopsy (diagnose, may be needed if none of these are present)	Characteristic cysts plus skin biopsy confirming fibrofolliculomas; *FLCN* gene mutation	Characteristic radiologic findings with history of cigarette smoking; histopathologic confirmation with CD1a- and CD207(langerin)-positive cell aggregates; often with other smoking-related changes; sequencing for MAPK pathway mutations	Autoimmune serologies or other corroborative testing to identify underlying condition. Lung biopsy may be needed in some cases	Diagnostic confirmation requires identification of organisms in sputum or bronchoalveolar lavage fluid. Detect Monoclonal antibodies
Complications		Recurrent spontaneous pneumothorax and chylothorax, progressive respiratory failure	Recurrent spontaneous pneumothorax, renal cancer	Pneumothorax, progressive respiratory failure, pulmonary hypertension	Progressive respiratory impairment, pneumothorax, rarely transforms to lymphoma	Progressive respiratory failure (similar to ARDS)
Management considerations		Serial lung function monitoring; bronchodilators; mTOR inhibitors for moderate to severe or progressive lung disease, or problematic chylous effusions, or large AMLs; early pleurodesis for pneumothorax; lung transplantation for end-stage disease	Early pleurodesis for pneumothorax; longitudinal renal cancer monitoring; family screening; premarital and prenatal guidance	Smoking cessation (the first-line treatment); bronchodilators ± ICS if airflow obstruction; vasodilators if pulmonary hypertension (not well established); seasonal influenza, pneumococcal, and COVID-19 vaccinations; serial lung function monitoring; systemic treatments: Cladribine or cytarabine; BRAF and/or MEK Inhibitors; lung transplantation	Serial lung function monitoring; treatment aimed at underlying disease (eg, immunosuppression for autoimmune disorders, antiretrovirals in HIV, immunoglobulin replacement in CVID)	Acute infections are treated with TMP-SMX, combined with corticosteroids in moderate to severe cases; the same regimen is also used for prophylaxis
Key references		13	75	59	76	77

Abbreviations: AML = angiomyolipoma; ARAF = A-Raf proto-oncogene, serine/threonine kinase; ARDS = acute respiratory distress syndrome; BRAF = B-Raf proto-oncogene, serine/threonine kinase; CNS = central nervous system; CVID = common variable immunodeficiency; FDS = folliculin deficiency syndrome; FLCN = folliculin; GGO = ground-glass opacity; HRCT = high-resolution CT; ICS = inhaled glucocorticosteroid; KRAS = Kirsten rat sarcoma viral oncogene homolog; LAM = lymphangioleiomyomatosis; MAP2K1 = mitogen-activated protein kinase kinase 1; MAP3K1 = mitogen-activated protein kinase kinase kinase 1, E3 ubiquitin protein ligase; MAPK = mitogen-activated protein kinase; MEK = mitogen-activated protein kinase kinase; MMPH = multifocal micronodular pneumocyte hyperplasia; mTOR = mechanistic target of rapamycin; NRAS = neuroblastoma RAS viral (v-ras) oncogene homolog; PH = pulmonary hypertension; PJP = *Pneumocystis jirovecii* pneumonia; PLCH = pulmonary Langerhans cell histiocytosis; RA = rheumatoid arthritis; SEGA = subependymal giant cell astrocytoma; SLE = systemic lupus erythematosus; TMP-SMX = trimethoprim-sulfamethoxazole; TSC = tuberous sclerosis complex; VEGF = vascular endothelial growth factor.

## From current challenges to future directions in imaging of DCLDs

### Imaging diagnostic uncertainties and technical limitations

The diagnosis of DCLDs is challenging due to CT artifacts and the considerable heterogeneity and overlap of cystic imaging features. While imaging may suggest certain patterns, definitive classification usually requires integration with clinical, genetic, and laboratory data. Chest radiography, while widely accessible and rapid, has low sensitivity for small or early cysts, cyst content evaluation, and differentiation from mimics, particularly in children.^78–82^ HRCT remains the cornerstone but is imperfect: small subcentimeter cysts are obscured by partial-volume effects, subjective and labor-intensive quantification hampers longitudinal comparison, cumulative radiation constrains serial surveillance, and limited spatial resolution for subtle disease is not fully compensated by post-processing.^83^ Chest radiography lacks sensitivity for early lesions and cannot characterize cyst content, especially in children.^78^^,^^81^^,^^82^ Although ultrasound provides some value in complication assessment and submerged visualization via acoustic windows, its overall role in DCLDs is restricted by low sensitivity for vascular supply, limited penetration, operator dependency, and anatomic blind spots.^84–87^ MRI avoids ionizing radiation and can integrate structural and functional data, but its role is compromised by respiratory motion artifacts, low pulmonary proton density, subcentimeter resolution constraints, long acquisition times, high cost, and restricted availability of ultra-short echo sequences. Positron emission tomography (PET)-CT adds metabolic information but suffers from nonspecific fluorodeoxyglucose (FDG) uptake in both neoplastic and inflammatory or infectious cystic lesions, while low-grade tumors may remain undetected.^88^

### Emerging imaging techniques and future opportunities

To address these challenges mentioned above, several innovations are on the horizon. Photon-counting detector CT with 1024-matrix reconstruction enhances visualization of sub-millimeter small airway walls without additional radiation, enabling early structural change detection.^89^ MRI has overcome historical limitations through innovations such as low-field systems, which reduce air-tissue susceptibility artifacts, and ultra-short-echo (UTE) sequences that capture signals from short-T2 tissues, improving parenchymal and cyst wall resolution as thin as 0.5 mm.^90^^,^^91^ Functional MRI techniques, including hyperpolarized gas MRI (^3^He/^129^Xe), quantify ventilation defects and alveolar microstructure, detecting early disease in DCLD even with normal pulmonary function.^92^^,^^93^ Oxygen-enhanced MRI maps regional ventilation-perfusion mismatch, correlating with spirometric parameters like FEV_1_.^94^ Prenatal/fetal MRI already surpasses ultrasound in discriminating congenital cystic malformations by vascular origin and morphology.^95^ AI enhances diagnostic precision through automated cyst detection and segmentation on HRCT, achieving highly accuracy and better temporal consistency.^96^^,^^97^ AI-driven radiomic signatures achieve 85% accuracy for etiologic discrimination of DCLDs.^98^ Integrative models that combine imaging, biomarkers, and clinical variables have the potential to stratify risk and forecast treatment response in patients with DCLDs.^99–101^ Emerging molecular imaging probes promise lesion-specific characterization of DCLDs within inflammatory or neoplastic milieus. Collectively, while current imaging modalities remain indispensable, their limitations necessitate a paradigm shift toward integrative, high-resolution, and intelligent strategies that convert subjective pattern recognition into quantitative biomarkers, enhancing diagnostic precision and optimizing patient care.

## Conclusion

In the precision-medicine era, imaging underpins not only diagnosis but also prognosis, risk stratification, and therapy monitoring in DCLDs. Within a comprehensive clinicoradiologic and multidisciplinary framework, emerging technologies, such as photon-counting CT, AI-driven analytics, and molecular imaging, can refine, not replace, this framework by enabling earlier detection and sharper phenotyping. Current evidence is limited by heterogeneous cohorts, regional bias, and short follow-up; therefore, large-scale multinational registries with standardized cyst annotations are now required to convert these technical advances into measurable patient benefit. Radiologists must transcend traditional image interpretation, promote integrated, multidisciplinary care, and champion these initiatives to translate technological promise into improved patient outcomes.
